# Numerical Analysis of Urea to Ammonia Conversion in Automotive Selective Catalytic Reduction Realistic Conditions

**DOI:** 10.1021/acs.iecr.1c02627

**Published:** 2021-09-23

**Authors:** Raúl Payri, Gabriela Bracho, Pedro Martí-Aldaraví, Javier Marco-Gimeno

**Affiliations:** CMT—Motores Térmicos, Universitat Politècnica de València, Valencia 46022, Spain

## Abstract

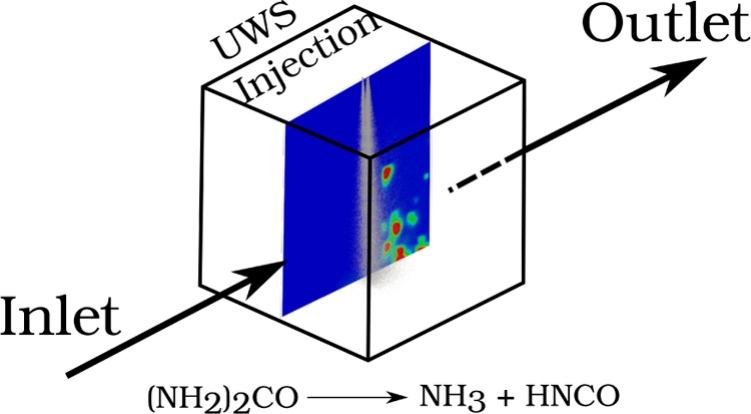

The selective catalytic reduction (SCR) is a technology employed for NO_*x*_ reduction purposes which is based on the injection of an Urea Water Solution (UWS) into the exhaust line. Conversion of this injected urea into ammonia is a key step to ensure high SCR efficiency. In order to study this phenomenon, a three-dimensional model of the urea–water injection process has been created to recreate realistic conditions. A Lagrangian–Eulerian approach has been followed to model liquid and gas phases, respectively. Droplet evaporation as well as relevant chemical processes have been included to recreate the thermolysis and hydrolysis phenomena, and the results have been validated against literature data. Then, the validated model has been applied to recreate an in-house experimental facility that measured spray macroscopic and microscopic characteristics by means of diffused back illumination (DBI) visualization. Probability density functions of the UWS droplet sizes as well as the velocity distributions have been obtained at three different regions of interest to be compared with the experimental data set. Contours of isocyanic acid and ammonia mass fractions have been included to show the chemical transformation from urea into its products. The model accurately replicates the experimental results, and it stands as a good methodology to predict the main spray characteristics as well as the chemical processes that take place in actual SCR systems.

## Introduction

The rise of the amount of vehicles for transportation purposes in the past decades has increased the awareness of the emission of combustion products to the atmosphere that are dangerous for human health.^1^ Nitrogen oxides or NO_*x*_ is one of the substances generated during the fuel combustion and thus the emissions are not only from diesel engines^2^ but also from novel carbon-free fuels such as ammonia.^3^ In order to prevent its introduction into the atmosphere, some technologies have been developed for this purpose that act in the combustion chamber prior to generating the nitrogen oxides, such as the variable valve actuation-based combustion strategies^4^ or in the exhaust pipes. One of the technologies that act on the generated NO_*x*_ is the selective catalytic reduction (SCR) which abates the NO_*x*_ into nitrogen and water by introducing ammonia prior to the catalyst. For safety and toxicological reasons, a urea water solution (UWS) is preferred instead of directly ammonia as a reducing agent.^5^ The injected urea subjected to the exhaust high temperatures suffers from thermolysis (eq 1) and the isocyanic acid (HNCO) undergoes hydrolysis (eq 2) transforming into ammonia.^6^
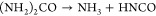
1
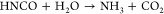
2

In the catalyst, the exhausted NO_*x*_ in combination with ammonia is decomposed into H_2_O and N_2_.^7^ Therefore, the chemical processes that urea undergoes to transform into the NO_*x*_ reducing agent needs to be properly understood. An inappropriate mixing with the surrounding air and incomplete evaporation before entering the catalyst could lead into low SCR efficiencies. To ensure a proper SCR behavior, enough residence time and flow turbulence should be provided to obtain a homogeneous NH_3_ distribution.^8^ However, the spatial limitation of such systems, as well as the transient behavior of the Internal Combustion Engines (ICE) implies that if it is not properly designed or controlled, the spray could impinge into the exhaust pipe walls creating liquid films that transform into solid deposits of urea byproducts.^9,10^

Kim et al.^11^ worked both experimentally and computationally on the analysis of chemical reactions in UWS for SCR. Spray mixing and thermal decomposition of UWS were the scopes of interest. Aqueous solution was injected at a marine diesel engine exhaust designed to provide the same flow rate and temperature of diesel engines and ammonia concentration was measured at different points of the line. The associated computational work was focused on replicating the experimental facility and validating the model. Diameter distributions of the injected solution were also measured, obtaining the proper Rosin–Rammler coefficients. Macroscopic results such as the spray width were also obtained. The computational model validation was performed by comparing the ammonia concentration at the very same locations than the experimental facility, showing a good agreement between the results. Birkhold et al.^12^ modeled the evaporation of a single droplet of UWS by means of a rapid mixing (RM)^13^ and a diffusion limit (DL) model.^14^ Due to the lower vapor pressure of the UWS compared to pure water, slower evaporation rate was found, and a continuous increase in the droplet temperature. Some differences were found in the droplet surface concentration between the RM and DL models, but a general agreement was found. The RM model was extended to a computational fluid dynamics (CFD) simulation, and the results were validated against Kim et al.^11^ results, revealing that a complete UWS evaporation and decomposing is not achieved in real configurations, especially at temperatures below 573 K where no significant hydrolysis is reported. Optical facilities were employed by Tang et al.^15^ to visualize and assess the formation of solid deposits at low exhaust temperatures. The urea decomposition was investigated by means of a FTIR (Fourier transform infrared) analyzer. The urea decomposition showed great dependency with the exhaust gas temperature as half of the urea did not decompose at 673 K. Below 513 K, the 40% deposits formed at the tailpipe did not decompose, and only above 873 K did the residual mass totally disappeared. Ebrahimian et al.^16^ developed evaporation and kinetic sub-models in order to describe the evolution of the main reaction products as well as the byproducts. Their results were compared with the experimental data in order to validate the presented model. Urea has an effect on the evaporation of water, as well as the UWS temperature. Reducing the gas temperature and hence reducing the heating rate result in increasing the direct decomposition pathway (hydrolysis) at the expense of a decrease of the polymerization pathway (thermolysis of the HNCO into NH_3_) due to the higher activation energy of the latter. In the line of the related computational work, Luo et al.^17^ employed a detailed kinetic urea decomposition model, while a surface chemistry model^18^ was employed on the SCR region. A steady-state simulation was performed and compared to a transient simulation, which sped up considerably the simulation and obtained accurate results. The position of the mixer was also of importance according to Luo et al., when located further from the SCR, the uniformity index was increased and consequently the NO_*x*_ conversion rate increased as well. Regarding the work performed to assess the NO_*x*_ conversion to non-harmful products, Rajesh Chundru et al.^19^ developed a Kalman filter estimator to asess the internal states of the SCR, predicting internal conditions within 5% of the experimental data employed. Pla et al.^20^ created a model capable of estimating the NO_*x*_ and NH_3_ emissions after the SCR based on an extended Kalman filter, evaluating it on standard conditions and with urea injection failure events, improving the prediction of the NO_*x*_ and NH_3_ slip on all conditions.

The above-mentioned studies did focus on the chemical phenomenon that takes place within the exhaust pipe and analyzed the conversion efficiency both of urea to ammonia and of the NO_*x*_ reduction processes. Information regarding the effects of the chemical reactions on the spray macroscopic characteristics is needed to further understand improvement mechanisms of the SCR system. Therefore, the main goal of this work consists of characterizing the spray and chemical processes of a UWS injection. For it, an appropriate urea to ammonia model will be constructed in order to apply it on a computational injection of UWS in an injection chamber. To the best of our knowledge, there is no previous work analyzing the differences between an inert and chemical model, as well as assessing the two main chemical reactions that the UWS undergoes to transform into ammonia.

This article will be divided into the following sections. First of all, the topics of interest have been introduced in this first part. After that, the methods employed to carry out this work are described, including the validation of the chemical model needed to perform the remaining simulations. That section will be followed by the description of the main results obtained, and the conclusions extracted from the study will close up the document.

## Methods

### Governing Equations

Simulations have been performed using commercial CFD software CONVERGE 3.0. A discrete droplet model (DDM) has been used to model the gas and liquid phases within an Eulerian–Lagrangian framework. The flow dynamics are controlled by the transport equations of mass (eq 3), momentum (eq 4), energy (eq 5), species (eq 6), and turbulence are derived from Navier–Stokes expressions. The droplets of the liquid phase are introduced as parcels, which represent a set of drops with identical characteristics such as velocity, temperature, diameter, and so forth. Their motion is controlled by eq 7.
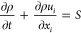
3
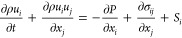
4
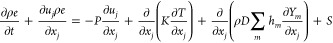
5
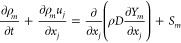
6
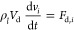
7

From the previous equations, ρ stands for the flow density, *u*_*i*_ is the velocity vector, *t* represents the time, and *S* stands for the source term, while *x*_*i*_ represents the spatial coordinates. In eq 4, *P* represents the pressure variable, while σ is the viscous stress tensor. With respect to eqs 5 and 6, *e* is the specific internal energy, *D* is the mass diffusion coefficient, *K* is the conductivity, *h*_*m*_ is the species enthalpy, and *T* is the temperature. Regarding eq 7, ρ_l_ is the liquid density, *V*_d_ is the droplet volume, *v*_*i*_ is the droplet velocity, and *F*_d,*i*_ stands for the sum of drag and gravitational body forces.

When it comes to the turbulence modeling, both for the validation and objective simulations, a Reynolds–Averaged Navier Stokes (RANS) approach has been taken, which decomposes the presented transport variables into their mean and fluctuating components. From it, the two equation *k*–ϵ RNG models have been used for the modeling of the structures that may take place within the geometry as it has been previously employed for low injection pressure applications.^21^ With it, the turbulence length scale is defined by eq 8, and the whole Reynolds stress tensor is defined by eq 9. From these equations, *C*_μ_ is a model constant, ε is the turbulent kinetic energy dissipation, *k* is the turbulent kinetic energy, μ_t_ stands for the turbulent viscosity, and *S*_*ij*_ is the mean strain rate tensor.
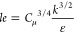
8
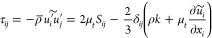
9

Regarding the injection process, several models have been included to properly represent the spray. The droplet no time counter (NTC)^22^ model has been activated to consider a coalescence interaction between the liquid particles, as the UWS spray is to be injected through three coplanar orifices, a droplet–droplet interaction is expected. Previous SCR-related studies showed influence on the droplet diameter distribution when the collision model was activated^23^ when using a flat headed six-hole injector. The primary and secondary breakup phenomena have been modeled thanks to a Kevin–Helmholtz Rayleigh–Taylor (KH-RT) model.^24^ The primary breakup is expected to happen on the injected blobs whose size is comparable to one of the injector, as they represent liquid ligaments. On the other hand, the Webber number associated with the typical injection pressures of such fluids is below 12,^25^ which implies that a secondary breakup is not expected.^26^ When it comes to the evaporation phenomena, the phase change of water is modeled by the Frossling correlation,^27^ approximating the droplet radius change rate by eq 10, based on the scaling factor for the mass-transfer coefficient (α), the mass diffusivity of liquid vapor (*D*), and the Sherwood number, *T*
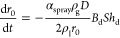
10

With respect to the thermolysis procedure (eq 1), the rate of generation of NH_3_ and HNCO is based on the urea rate of degradation and evaporation. The temperature change is calculated by two means depending on the droplet diameter.^28^ Large droplets (>100 μm) use a spherically symmetric heat relation (eq 11), based on the thermal conductivity (*k*_d_), the distance to the droplet center (*r*), the change in enthalpy due to urea decomposition (*H*_decomp_), and the convection coefficient between the surrounding gas and the droplet (*h*). Small droplets (<100 μm) on the other hand follow eq 12 to compute the same temperature rate of change in which the temperature distribution within the droplet is assumed to be uniform.
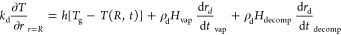
11
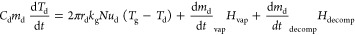
12

The decomposition of urea is driven by an Arrhenius correlation (eq 13) which computes the rate of change of the radius by means of a factor, the activation energy, the density of urea, and the droplet temperature.
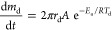
13

On the other hand, hydrolysis is computed by means of the SAGE chemical kinetic solver.^29^ The set of ordinary differential equations is solved by the CVODE solver.^30^ The NH_3_ reaction rate was defined according to eq 14, where *q*_r_ is the rate of progress of the reaction and *v*_m,r_^′^ and *v*_m,r_^″^ are the stoichiometric coefficients of the reactants and products, respectively. The mass and energy conservation equations result as indicated in eqs 15 and 16.
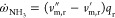
14
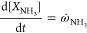
15
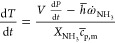
16

### Model Validation

**Figure 1:**
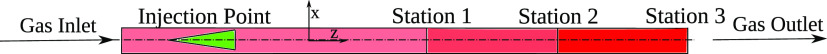
Schematic view of the validation geometry, and the corresponding ammonia measuring station.

The described model has been validated against experimental results from Kim et al.^11^ The geometry, which consists of a cylinder with a diameter-to-length ratio of 0.046 and a diameter of 0.3 m, has been recreated (Figure 1). The injector is placed axially in the center of the previous geometry at a distance of 0.5 m of the cylinder inlet, and a UWS with mass fractions of 60% of water and 40% of urea is injected. A constant injection pressure of 14 bar has been set. The validation of the chemical model has been performed at all the incoming gas velocities and temperatures that the original experiment was tested at, and they are summarized in Table 1.

**Table 1:** Set of Gas Temperature and Gas Velocity Conditions in Order to Validate the Chemical Model

gas temperature (K)	gas veloc. 1 (m s^–1^)	gas veloc. 2 (m s^–1^)	gas veloc. 3 (m s^–1^)
673	10.8	8.3	6.0
623	10.8	9.1	6.4
573		9.0	6.6

A base size element of 0.03 m has been selected after performing a mesh sensitivity analysis, resulting in a base cell number of 150,000 elements, and the adaptive mesh refinement (AMR) has been activated to detect where strong velocity and density gradients are located and refine the mesh size according to it, up to two refinement levels, which implies a maximum cell count during a simulation of 1 million. Therefore, the minimum cell size introduced will follow eq 17, where *p* is the number of refinement levels introduced. The simulations have been run until a steady-state solution is achieved, which has been assessed by analyzing the amount of ammonia that is created at the three measuring sections (depicted in Figure 1), as it is the main parameter of interest for the later study. The conversion efficiency has been defined by calculating the ratio of ammonia obtained by the simulations to the theoretical amount of ammonia that would have been obtained if all the urea injected had converted to ammonia, obtained by eqs 1 and 2. This parameter has been calculated at the measuring sections located at 3.0, 4.5, and 6.0 m with respect to the injection point location.
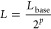
17

**Figure 2:**
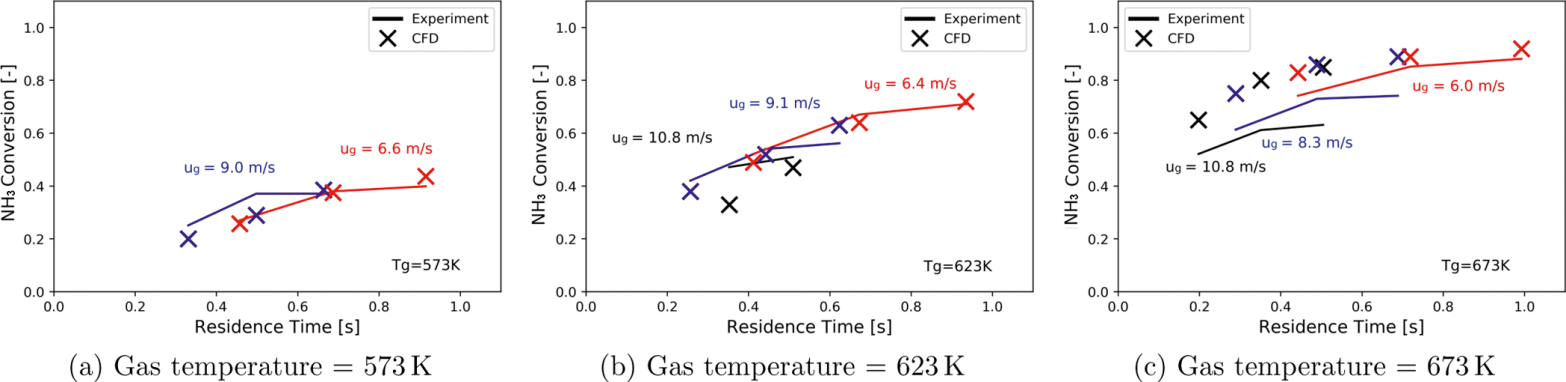
Urea to ammonia conversion efficiency for the three gas temperatures and the different incoming gas velocities simulated.

The results are shown in Figure 2 for the whole simulation matrix at the three measuring stations. The residence time has been calculated based on the incoming gas velocity in order to distinguish between the mentioned measuring positions within the pipe, following the same criteria as in the experiments of Kim et al.^11^ There is a general good agreement on the conversion efficiencies at low gas temperatures (Figure 2a), while for high gas temperatures (Figure 2c), discrepancies up to 20% can be seen. Both thermolysis and hydrolysis models could be adding uncertainties at higher temperatures where both play an important role. At lower temperatures (573 K), only degradation of urea via thermolysis happens, reducing those discrepancies. Nonetheless, the lower the exhaust gas velocity, the higher the agreement between the experimental and computational results. The full dependence on the temperature of the Arrhenius correlation for the urea degradation, and the lack of relationship with convective effects might be playing a role in this particular discrepancy.

**Figure 3:**
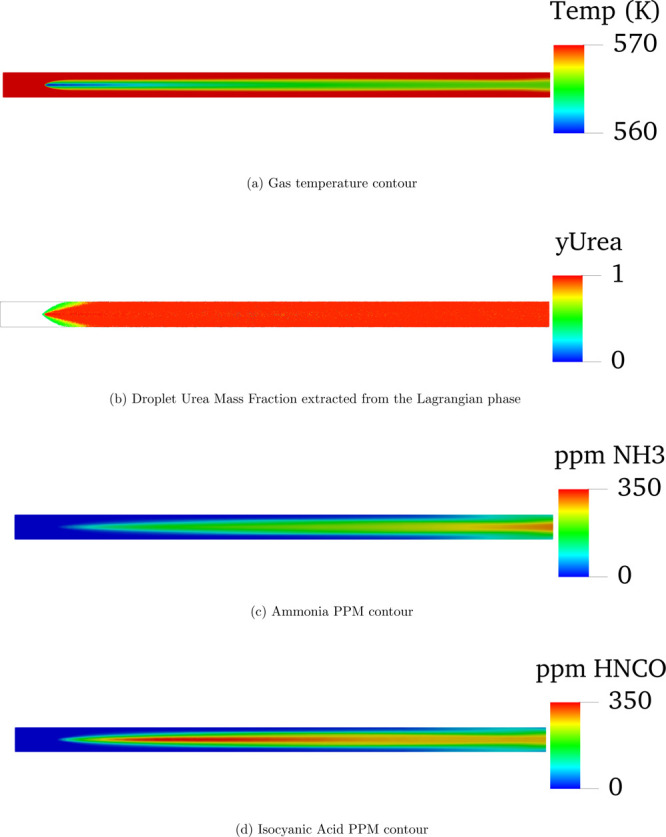
Ammonia and isocyanic acid PPM contours after the simulation has reached a steady state at 573 K and 6 m s^–1^ boundary conditions.

Figure 3 shows the different contour results for 573 K and an inlet gas velocity of 6 m s^–1^, being the simulation with the maximum gas residence time and a temperature above which chemical degradation of urea should be expected. The main temperature drop (Figure 3a) occurs at the center of the pipe as smaller droplets, which show a smaller radial penetration and evaporate and transform faster than the bigger ones, are located near the pipe center. The evaporation of water (Figure 3b) happens rather quickly in the first few instants after the injection. In agreement with the temperature contours, the evaporation of water in the small droplets is faster in the center of the pipe due to the presence of small droplets there. On the spray outskirts, the increase of urea mass fraction takes place later. The results from the thermolysis reaction are seen on Figure 3d, where a faster rise in the amount of HNCO right after the injection point is observed. According to eq 1, from one molecule of urea injected, a molecule of ammonia is created, and only after the generated HNCO appears, together with the evaporated water of the UWS, another molecule of ammonia appears (eq 2). Right after the hydrolysis starts taking place, the ppm of HNCO starts to slightly decrease. That is the reason why the maximum amount of ammonia is found at the outlet of the simulated geometry.

### Realistic Operation Conditions

**Figure 4:**
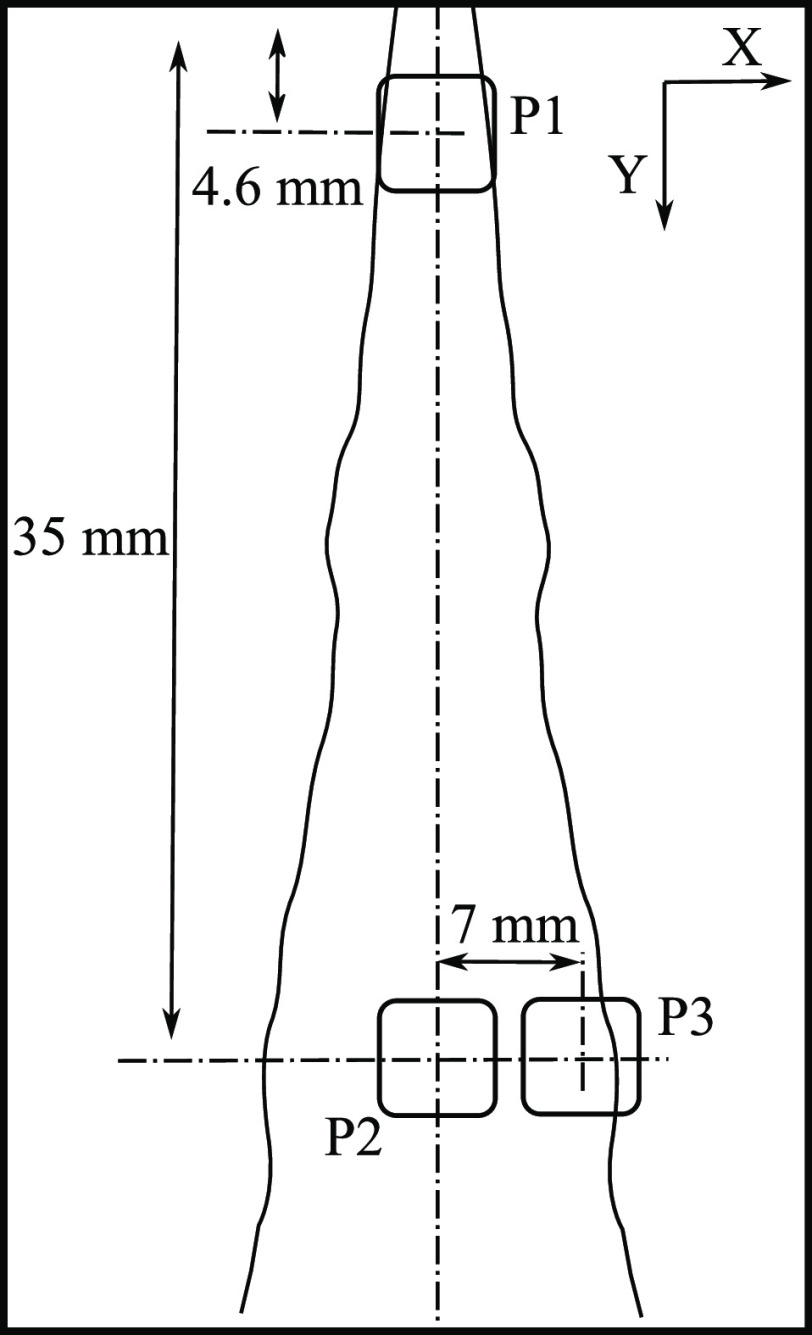
Windows of interest used to determine the PDF curves.^25^

Once the chemistry model is validated, it is then translated to a computational recreation of an in-house experimental test rig. The rig consisted of an injection chamber, whose dimensions are 70 mm × 70 mm × 180 mm. Thanks to an electric resistor, heated air is introduced at a certain flow rate into the injection chamber. The facility is capable of reproducing exhaust flow rates of up to 400 kg h^–1^ and gas temperatures of 400 °C, although both conditions cannot be met simultaneously. To compare the experimental injection results with the CFD data, a gas flow rate of 40 kg h^–1^ has been used. Gas temperatures have been set to 453 and 623 K. The UWS injector is located in the upper wall, and therefore, the UWS is injected perpendicularly to the incoming gases in contrast to the coaxial injected spray of the validation case. The injector is a Bosch dosing unit, consisting of three counter-sunk orifices of 145 μm of diameter. A detailed description of the mentioned facility has been provided by Payri et al.^31^ Three injection pressures have been tested (4, 6, and 8 bar) to cover the range of operation of such devices. The solenoid of the injector has been energized for 5 ms to inject 6 × 10^–6^, 7.2 × 10^–6^, and 8.2 × 10^–6^ kg of UWS for 4, 6, and 8 bar of injection pressure, respectively. In this case, the fluid mixture is composed of 67.5% H_2_O and 32.5% of (NH_2_)_2_CO. In order to predict probability density function (PDF) plots of droplet diameters and droplet velocities, a diffused back-light illumination (DBI) technique^32^ was set up. From within the injected spray, three specific regions of interest were defined to analyze the evolution of the PDF plots. These regions are illustrated in Figure 4, named P1, P2, and P3.

**Figure 5:**
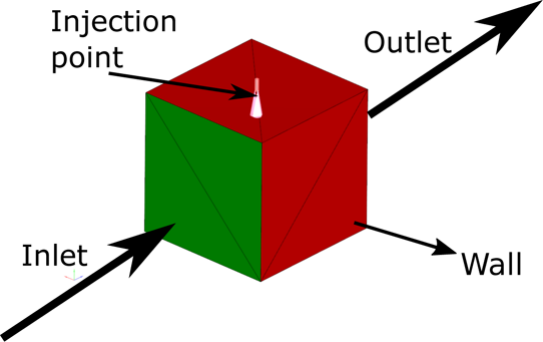
Computational domain employed to simulate the injection of the UWS.

**Figure 6:**
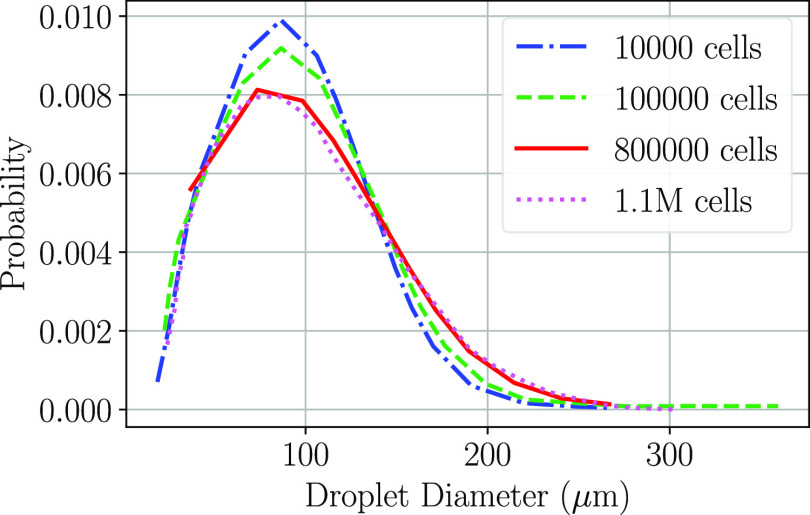
Mesh independence results performed for a simulation of 6 bar of injection pressure and an air temperature of 623 K.

**Figure 7:**
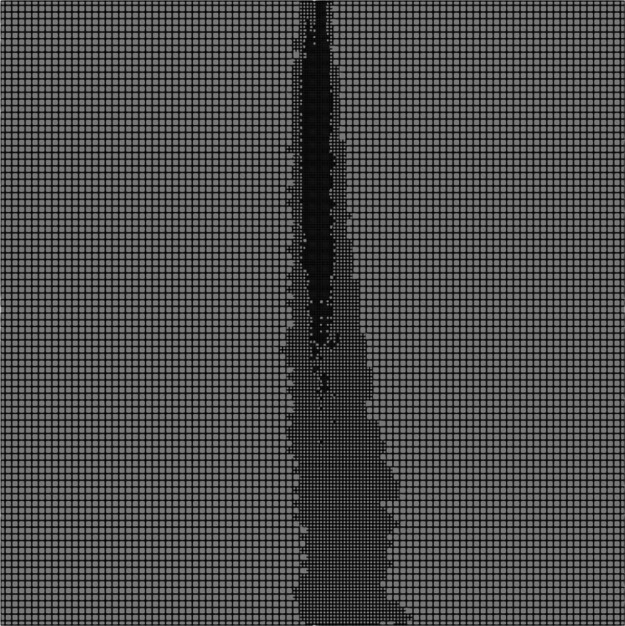
Mesh snapshot during the injection event.

In computational terms, the geometry employed in which the transport equations are to be solved is the one used by Payri et al.,^25^ which consists of a simplification of the experimental injection chamber, reducing it into a cube of 70 mm × 70 mm × 70 mm (Figure 5) without affecting the accuracy of the presented model. The computational geometry consists of two surfaces that act as the velocity inlet and pressure outlet boundary conditions, respectively. The remaining surfaces act as the wall boundary conditions. The UWS flow rate profile obtained by experimental means^31^ has been applied to the computational model. In the CFD simulations as in the experiments, the spray is injected within a steady and developed transversal gas flow, which has been initialized with a previous simulation performed at a gas flow rate of 40 kg h^–1^ and the corresponding gas temperature (453 K or 623 K). A sum up of the gas boundary conditions can be graphically seen in Figure 5 and Table 2. The droplet size distribution to be introduced into the computational domain is defined by a Rosin–Rammler probability distribution (eq 18). The distribution is controlled by the scale and shape parameters. In order to analyze the differences between the inert and the chemical simulations, the very same parameters have been chosen by Payri et al.,^25^ who found that the scale parameter was highly influenced by the inner geometry of the injector. Therefore, *k* = 3 stands for the selected shape parameter, while the scale parameter has been set to *d*_0_ = 0.3*d*_*n*_, where *d*_*n*_ is the nozzle diameter. The amount of parcels introduced to properly represent the UWS has been set to obtain a reference value of 1.5 × 10^–10^ kg of mixture per parcel.^33^ The mesh sensitivity study has also been carried out to determine the optimal mesh to resolve the presented problem. The base element size was set to 1.5 mm, which was increased and reduced for mesh independence purposes, concluding into a total cell count of 800,000 cells with a base size of 0.75 mm, which will possibly be increased by the AMR tool up to a minimum cell size of 0.19 mm, as it happened in the work of Payri et al.^25^ The results of this study are presented in Figure 6, and an example of the mesh is included in Figure 7, where an example of the AMR effects can be seen in the refined cells. The resolution in the near-wall region is not sufficient to properly resolve the boundary layer, as the *y*+ value of the first cell has a value of 30. Therefore, a law-of-the-wall model has been employed, assuming that the cell falls within the log-law region of the boundary layer. No spray–wall interaction has been set in the computational model, as the interest of this study is present prior to this event. Therefore, the droplets that impacted with the domain walls vanished, lightening up the simulations performed. A summary of the DDM model has been included in Table 3.
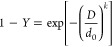
18

**Table 2:** Set of CFD Boundary Conditions Introduced for the Realistic Operating Conditions Simulations

parameter	value
inlet flow rate	40 kg h^–1^
inlet gas temperature	453 K, 623 K
pressure outlet	101,325 Pa
wall boundary condition	wall model

**Table 3:** Set of DDM Boundary Conditions Introduced for the Realistic Operating Condition Simulations

parameter	value
working fluid	commercial AdBlue
injection pressure	4–6–8 bar
injection profile	experimentally extracted
droplet distribution	RR *k* = 3, *d*_0_ = 0.3*d*_*n*_
injection excitation time	5 ms
injection temperature	300 K
breakup model	KH-RT
amount of parcels	4 million

## Results

### Droplet Size and Velocity Comparatives

**Figure 8:**
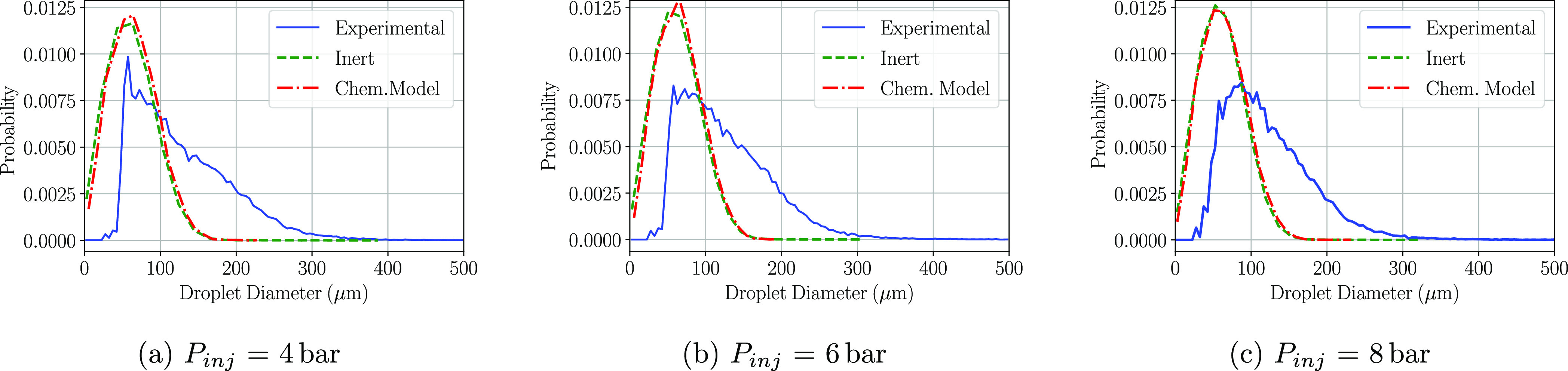
PDF distributions of the detected droplet diameters at the P1 region at a cross-flow gas temperature of 623 K.

**Figure 9:**
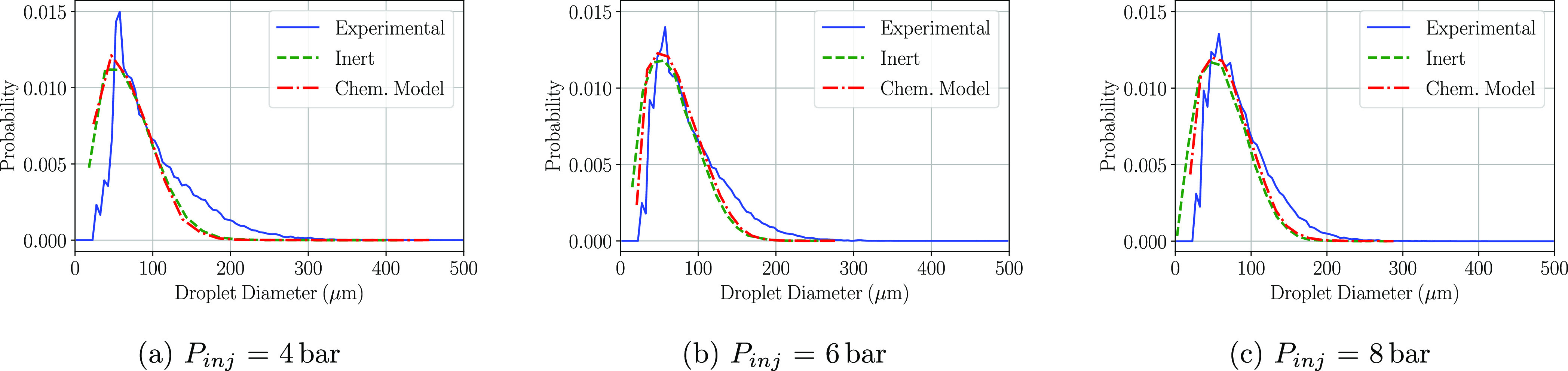
PDF distributions of the detected droplet diameters at the P2 region at a cross-flow gas temperature of 623 K.

**Figure 10:**
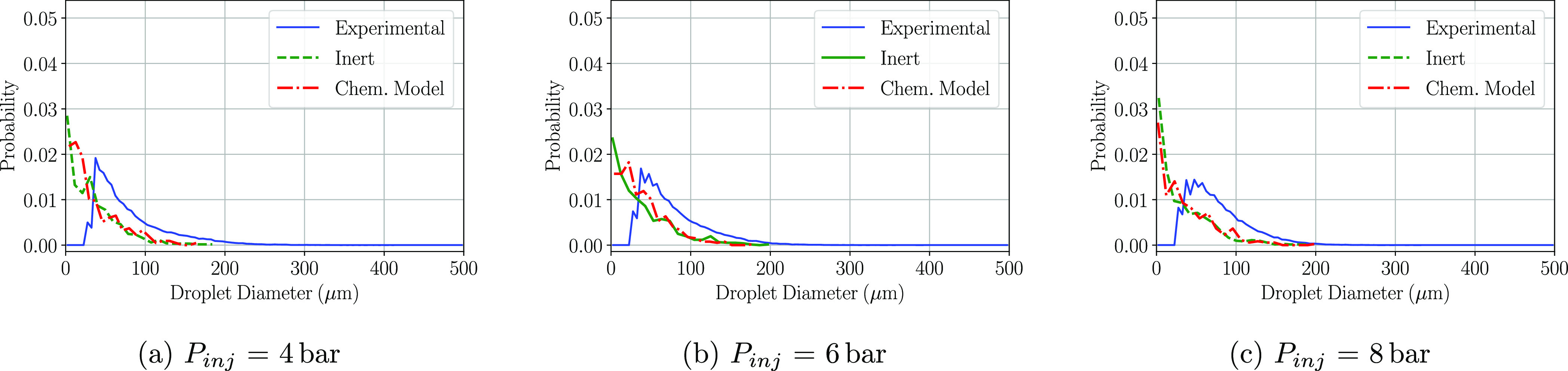
PDF distributions of the detected droplet diameters at the P3 region at a cross-flow gas temperature of 623 K.

The PDFs have been obtained for the three windows of interest described (Figure 4). Only the droplets obtained in a section with 1 mm of thickness centered in the *z*-axis have been considered to recreate the depth of field captured by a camera. The information regarding the droplets that go through the mentioned windows is gathered, and a histogram has been computed in order to later recreate the PDF plots. Figures 8–10 show the PDF of the droplet diameters at the three positions and at the three injection pressures simulated. At the immediate zone of the injector exit (P1), whether the chemical model or not was included, there is a droplet probability overprediction at small diameters. No differences between the computational models (inert and chemical) are observed at any of the injection pressures shown in Figure 8a–c, as the residence time of the injected droplets is not sufficient in any of the three pressures to undergo any kind of chemical reaction.

When it comes to the results obtained at P2, the matching of the PDF plots is accurate whether the urea degradation is implemented or not and for the three injection pressures tested. Little variation in the density functions is observed between the three conditions. The chemical model simulations show slightly higher differences with respect to the inert model if compared with the results of P1. The amount of droplets below 50 μm is lower, increasing the probability of finding droplets larger than 50 μm, which results in a similar diameter distribution compared to the experimental results in which some urea degradation phenomena should be expected.

Regarding the last window of interest, P3, including the transformation of urea into ammonia in the CFD simulations also overpredicts the amount of small droplets in all three injection pressures (Figure 10a–c). Thermolysis and hydrolysis effects start to be seen if compared both CFD curves. At low injection pressures (4 bar), the peak for the inert model is located at diameters smaller than 10 μm, while if the chemistry model is activated that probability peak is moved toward 20 μm. At higher injection pressures, 6 and 8 bar, the effect is not as significant; as for both cases, the probability peak location remains in the same droplet diameter and the probability of the bigger droplets is slightly higher.

Nonetheless, the differences that are observed by comparing the inert and chemical models are very subtle, which indicates that not enough water evaporation and thermolysis effects are taking place under these injection conditions.

**Figure 11:**
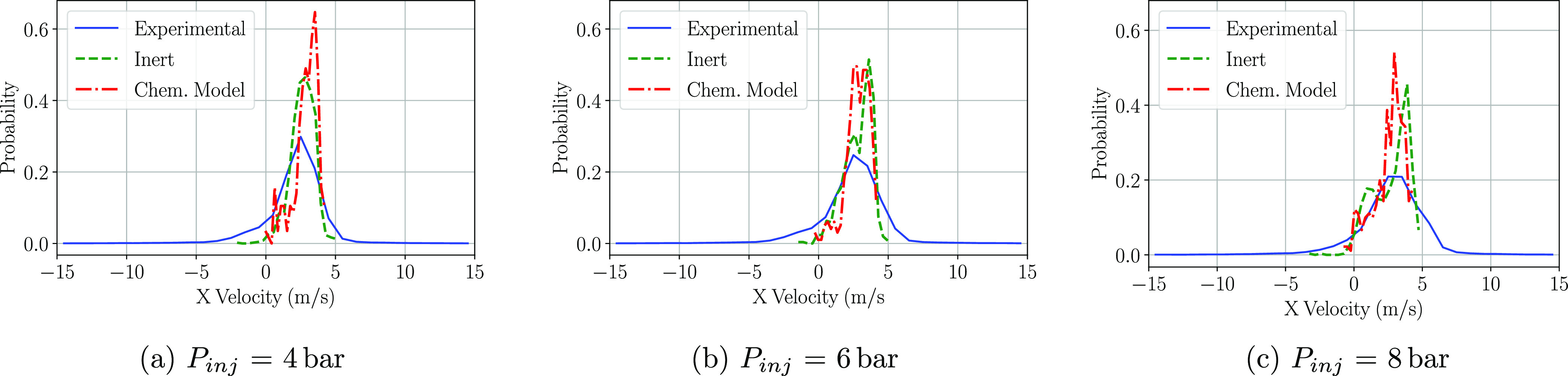
PDF distributions of the droplet *X*-velocities at the P3 region at a cross-flow gas temperature of 623 K.

**Figure 12:**
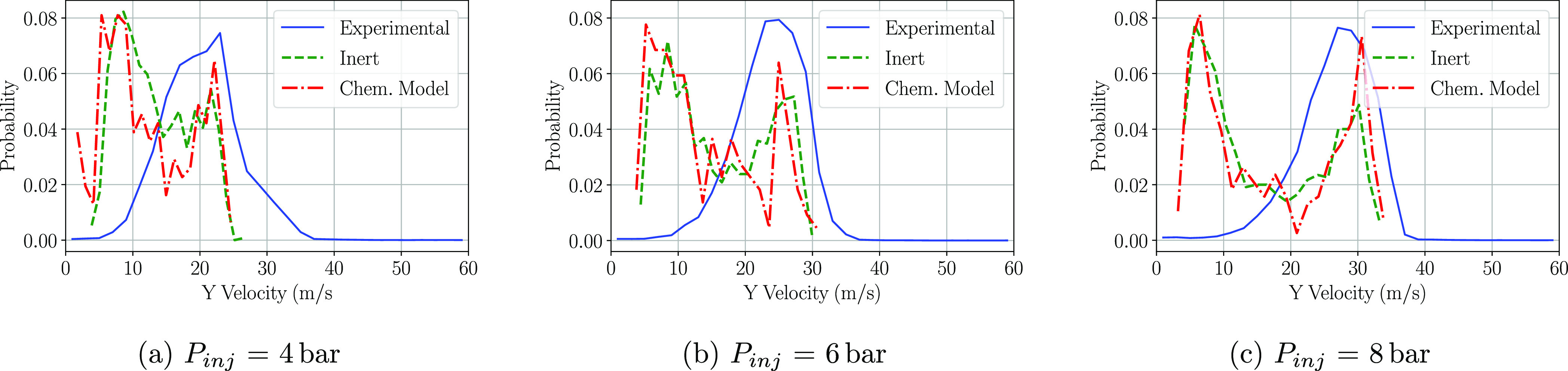
PDF distributions of the droplet *Y*-velocities at the P3 region at a cross-flow gas temperature of 623 K.

When it comes to the velocity distributions, no differences are detected at the P1 and P2 windows. Some differences are detected at the P3 window for both *X*-velocity and *Y*-velocity, and therefore, they are included in Figures 11 and 12. The peak *X*-velocity is matched at the three injection pressures and the trends as well. The higher the injection pressure, the wider the velocity distribution is, as the droplets that compose the spray outskirt do have a greater *X*-velocity. CFD also shows the same behavior as the probability peak is reduced with the increasing injection pressure. Computational methods overpredict the maximum calculated probability, and no significant differences are found within both inert and chemical models. Regarding the *Y* component of the velocity (Figure 12), greater differences arise with respect to the experimental results. The greater amount of low *Y*-velocity droplets is linked to the greater amount of droplets with a diameter smaller than 25 μm (Figure 10). These droplets modify their trajectory toward the domain outlet easier than larger droplets and therefore reduce the *Y*-velocity component. No significant differences arise between chemical and inert models as both mostly again agree on the distribution shape. This confirms that in the geometry and the simulation conditions employed there is almost no urea degradation phenomena due to the high similarity between the PDF curves.

**Figure 13:**
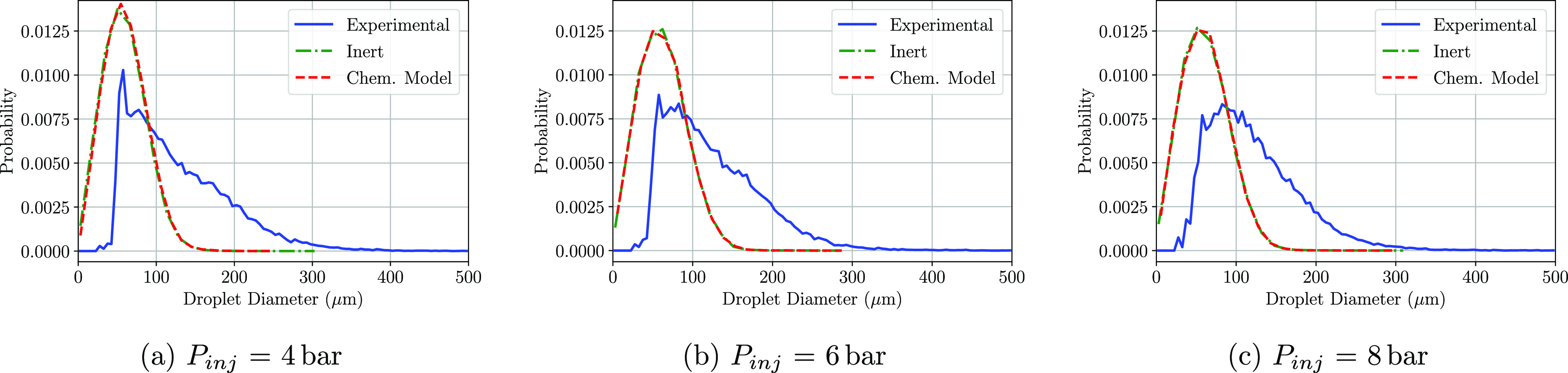
PDF distributions of the detected droplet diameters at the P1 region at a cross-flow gas temperature of 180 °C.

**Figure 14:**
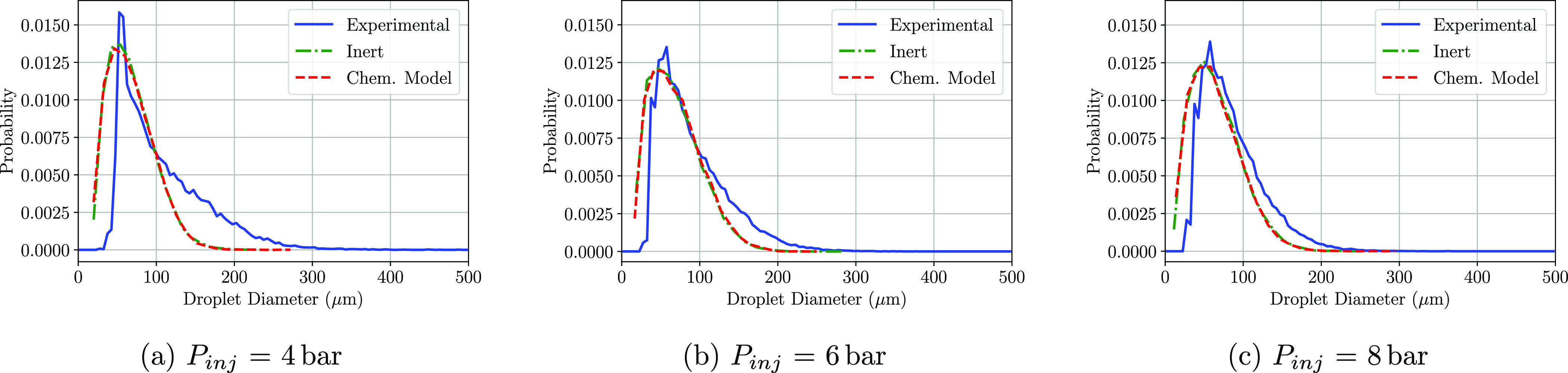
PDF distributions of the detected droplet diameters at the P2 region at a cross-flow gas temperature of 180 °C.

**Figure 15:**
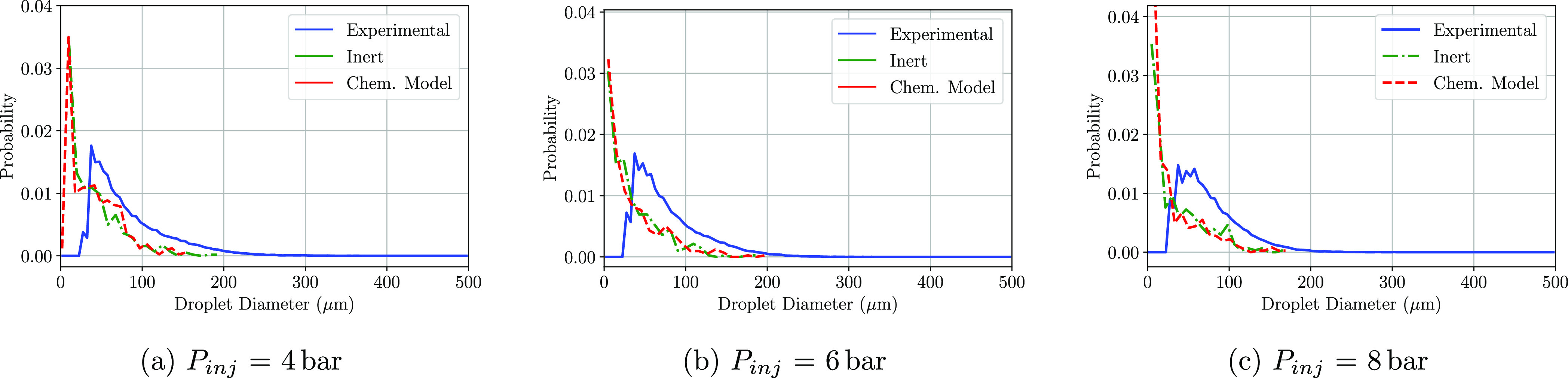
PDF distributions of the detected droplet diameters at the P3 region at a cross-flow gas temperature of 180 °C.

When it comes to the simulation performed at a lower gas cross-flow temperature (453 K), the corresponding PDF of the droplet size have also been calculated. The PDF results show agreement on the most frequent droplet diameter in P1 (Figure 13), although the CFD model overpredicts the probability as it happened for the simulations performed at 623 K. In P2 (Figure 14), a good prediction of the diameter distribution functions is found, as it happened for the 623 K case. Again, for P3 (Figure 15), an overprediction of droplets smaller than 50 m is present at the three injection pressures. At this particular temperature, no differences arise between the inert simulation and the chemical model introduced in all three windows of interest. This indicates that at the given gas temperature, almost no decomposition of the urea takes place. Considering also the results obtained for the validation case (Figure 2), the gas temperature therefore plays an important role on urea transformation into ammonia.

### Penetration of the Spray

**Figure 16:**
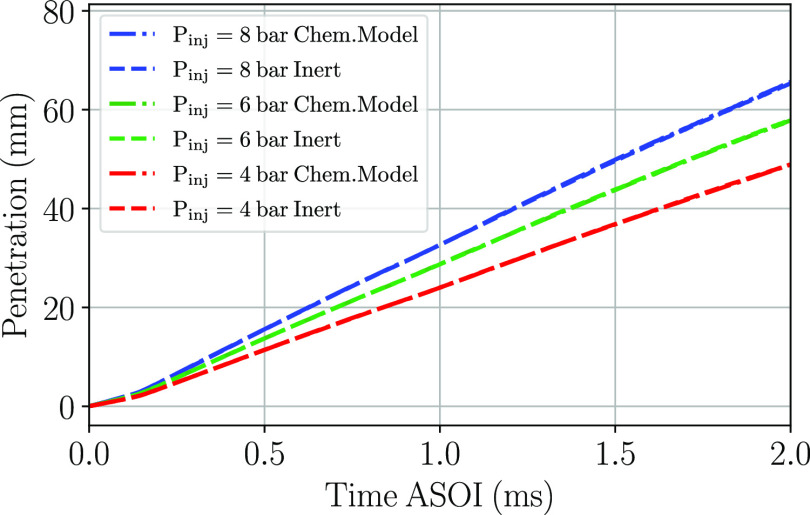
Penetration curves for the two computational models and the three simulated injection pressures for a cross-flow gas temperature of 623 K.

To further assess the differences between the chemical and the inert models, the penetration curves have been obtained at the three injection pressures simulated. The penetration has been defined according to the procedure described in the work of Payri et al.^25^ The results have been included in Figure 16. No differences are observed within the two computational models. The chemical model perfectly matches the penetration curves from the inert model. Therefore, no differences are expected between the penetration of both models at a gas temperature of 453 K. Expected differences in the curves are detected with the increase of the injection pressure. The higher the injection pressure the faster the spray tip travels and, therefore, the steeper the slope of the penetration curve is. The comparison with the experimental data set was already compared in a related work performed previously, whose results have already been published.^25^

### Sauter Mean Diameter Distribution

**Figure 17:**
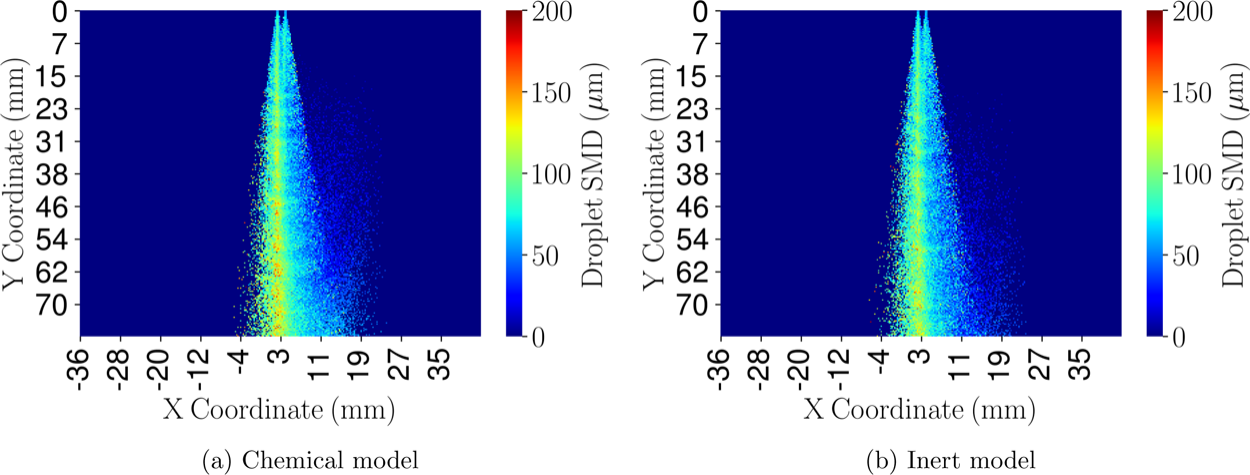
SMD contour comparison for both chemical and inert models at an injection pressure of 8 bar and cross-flow gas temperature of 623 K.

A lateral projection of the computational domain has been performed to analyze the spatial distribution of the spray. The computational geometry has been split into a grid with a resolution of 300 × 300 × 1 cells (*X*, *Y*, and *Z* directions, respectively, Figure 4). All the droplets that fall within each of these cells are collected, and a mean diameter is obtained. The Sauter mean diameter (SMD) has been chosen as the mean diameter found in each cell as it represents the ratio of the volume to the surface area of the set of droplets found. This projection has been computed for both computational models, and the results have been included in Figure 17.

Both plots agree on the main spray characteristics, the spray core is composed of larger droplets, above 120 μm, while the outskirts are made of smaller droplets. The momentum transferred by the cross-flow gases to the droplets wash away the smallest droplets. The larger droplets, which have a higher inertia, are not influenced that much by the gas momentum and therefore remain in the spray core. Nonetheless, subtle differences arise. The chemical model (Figure 17a) shows higher SMD values in the spray core region and shows a greater amount of tiny droplets that are being washed away by the incoming gases than in the inert model (Figure 17b). The presence of the model capable of degrading urea into ammonia implies that once water has evaporated, further diameter reduction happens due to thermolysis. As a consequence, the smallest droplets that are present in the core region evaporate, and the SMD of this region increases. On the other hand, a greater amount of small-sized droplets are present, which is easier to be dragged away by the incoming hot cross-flow gases.

### Droplet Diameter Change

**Figure 18:**
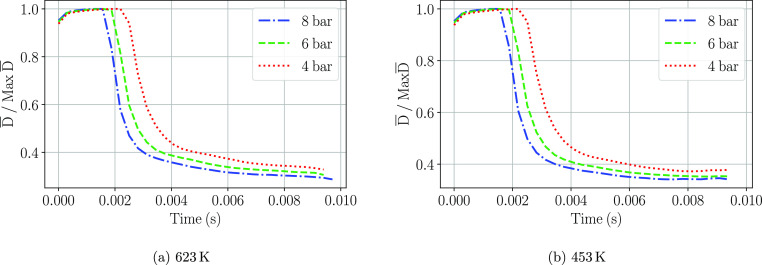
Evolution of the mean droplet diameter with time at the two gas temperatures.

Due to the little differences observed in the PDF plots of the droplet diameters injected, the chemical processes undergone have been assessed by obtaining the variation of the droplet diameter after they have been injected. To do so, each droplet diameter has been tracked throughout its lifetime during the simulation and they have been averaged. The results have been normalized by the maximum mean diameter value obtained. The obtained curves are presented in Figure 18.

Three specific regions can be detected from the mentioned plot. A rise of the droplet size is detected in the first instants after the start of the injection, a discrete size distribution is being introduced, and therefore, the maximum diameter appears some time after the start of injection. It rapidly reaches the maximum mean value when the droplet distribution injected is representative of the Rosin–Rammler curve introduced as an input parameter. After that moment, a rapid decrease of the diameter is detected, followed by a slower droplet size decrease. These two curve sections correspond to the water evaporation and the thermolysis reaction, respectively. The droplet breakup might contribute to the droplet radius decrease, although as already mentioned, it is not expected to happen, as it was not observed on previous work.^25^ The reaction enthalpy of the water evaporation (≈2300 kJ kg^–1^) is lower than the reaction enthalpy of the thermolysis process (≈3088 kJ kg^–1^).^12^ In addition, the heating up of the droplets during the evaporation of the water is translated into a lower heat transfer from the ambient gas toward the urea droplets. For the case of 623 K (Figure 18a), all injection pressures show the same evaporation slopes, but higher injection pressures (8 bar) start the water evaporation process earlier in time than the lower injection pressures (6 and 4 bar). As the evaporation rate is controlled by the steady-state relationship^26^ (eq 19) and correction factor (*C*) taking into account the convective and thermal effects^34^ (eq 20), which depends on the droplet Reynolds and Prandtl numbers, a faster penetration of the UWS spray into the hot gases enhances the local Reynolds number and hence the droplet vaporization rate. For the simulation with the cross-flow at 453 K (Figure 18b), the same three curve sections are found. The time instant where the droplet diameters start to decrease due to water evaporation matches the case of 623 K. Differences arise where the water has undergone complete evaporation, and thermolysis should be revealed in the curve. The slope of the curve is gentler for the 453 K case up to the point of seeing no further decrease in the mean droplet diameter, indicating that the surrounding gas temperature is a critical parameter for the urea decomposition. As reported by Yim et al.,^6^ urea only undergoes complete decomposition into NH_3_ at temperatures above 623 K.
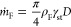
19
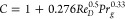
20

**Figure 19:**
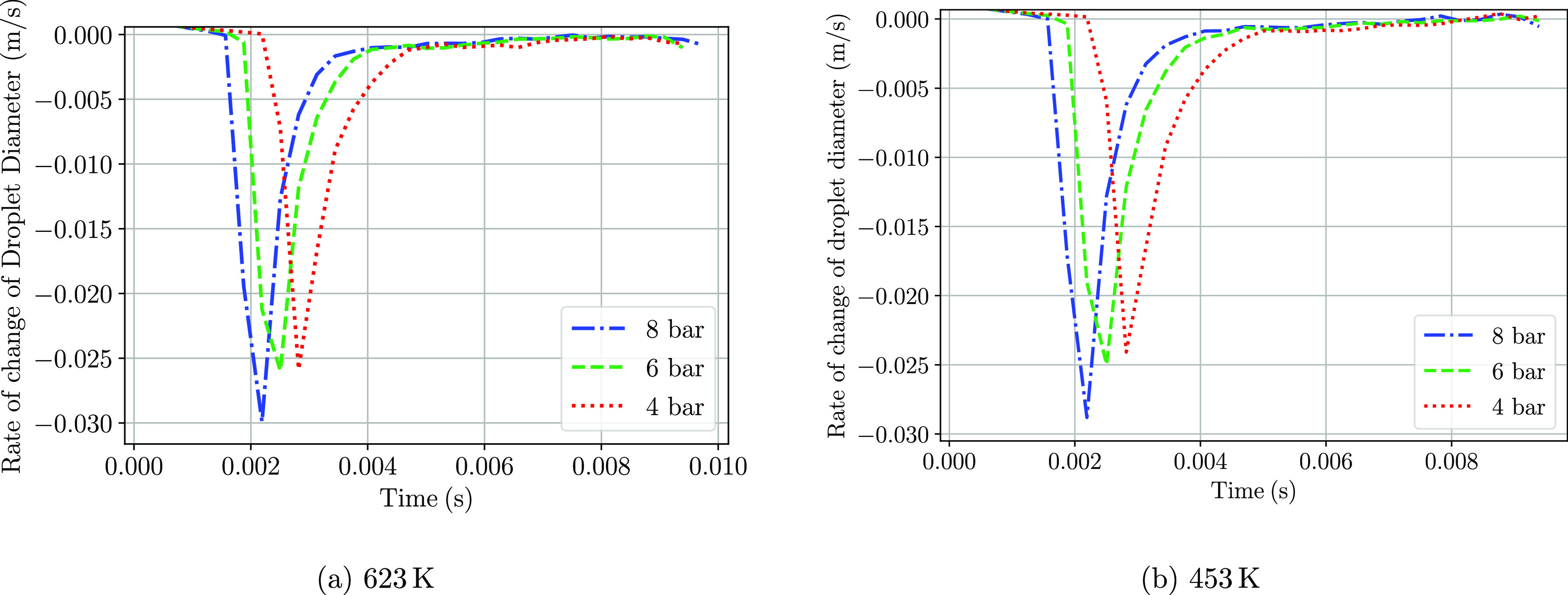
Rate of change of the droplet diameter during the simulation time for the two gas temperatures.

The derivative of the previous curves have also been computed to assess whether after the water evaporation, urea thermolysis takes place at both gas temperatures. The derivatives have been shown in Figure 19. In agreement to the droplet size evolution curves, the highest injection velocity shows the greatest rate of evaporation of the other two injection pressures. The same behavior is observed for the lower gas temperature case, although the maximum rates are slightly lower than that for the higher temperature simulation. Once the water has evaporated, the rate of evaporation rapidly decreases for both cases reaching almost a null value. Regarding the thermolysis region, no significant differences arise, although the 623 K case shows slightly higher rates.

### NH_3_ and HNCO Distribution

**Figure 20:**
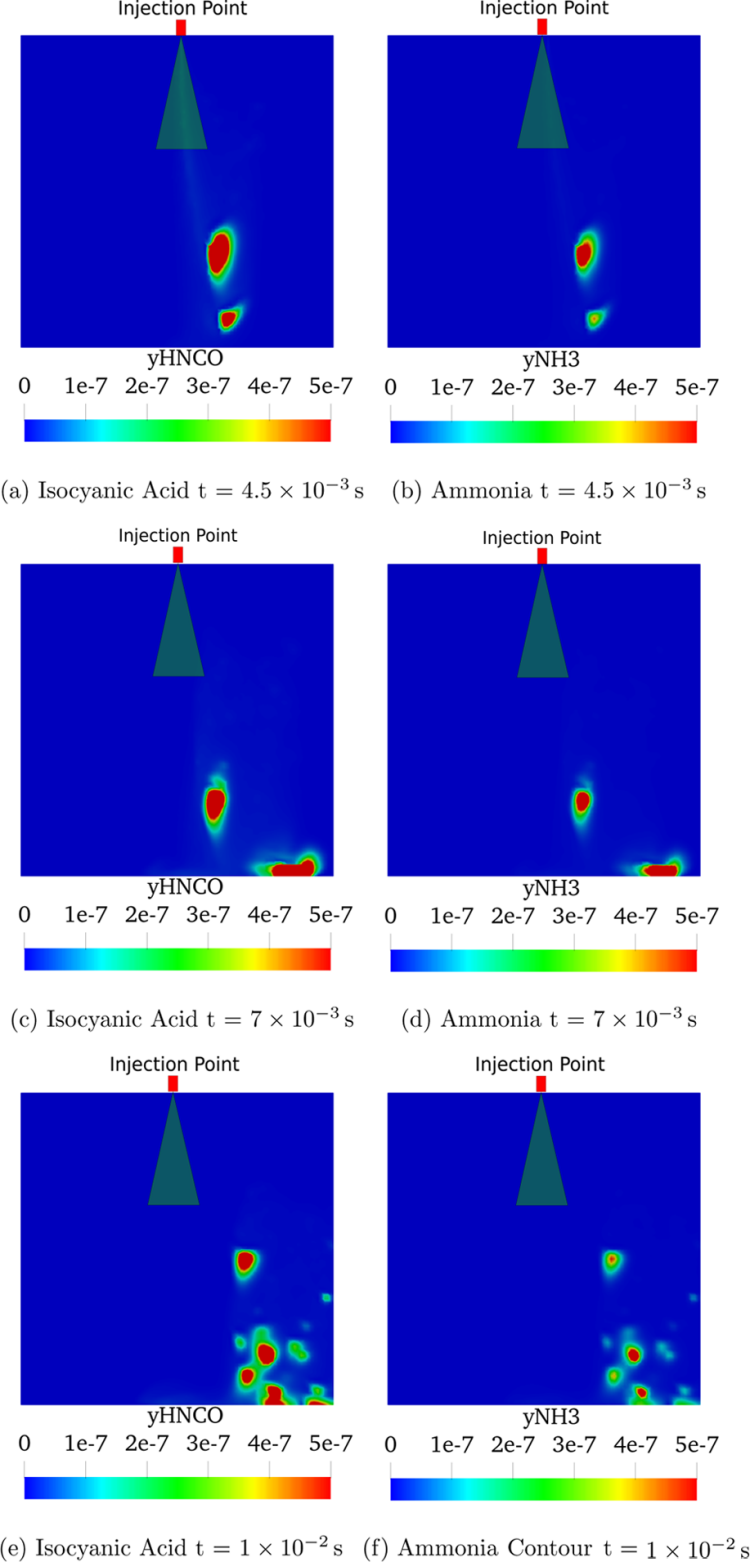
Ammonia and isocyanic acid mass fraction contours at a specific time instant for an injection pressure of 8 bar and 623 K of cross-flow gas temperature.

Isocyanic acid (HNCO) and ammonia (NH_3_) contours have been extracted for both cross-flow gas temperatures in order to confirm the lack of thermolysis reaction on urea. Figure 20 shows the results for the higher temperature case (623 K). In the first case, the generation of NH_3_ can be detected in isolated spots within the computational domain. These spots are located outside the spray cone being washed away by the incoming hot gases. The location of the ammonia spots match the coordinates where isocyanic acid is also being produced. This represents the thermolysis reaction (eq 1). The concentration of HNCO (Figure 20e) is lower compared to the amount of NH_3_ found (Figure 20f) due to the higher molecular weight of the HNCO compared to the NH_3_ one (*M*_NH_3__ = 17.031 g/mol, *M*_HNCO_ = 43.025 g/mol). At 453 K in concordance with the results observed by assessing the diameter curves (Figure 18), there is almost no presence neither of isocyanic acid nor ammonia gases, which allows stating that the degradation of urea does not occur at this gas temperature.

**Figure 21:**
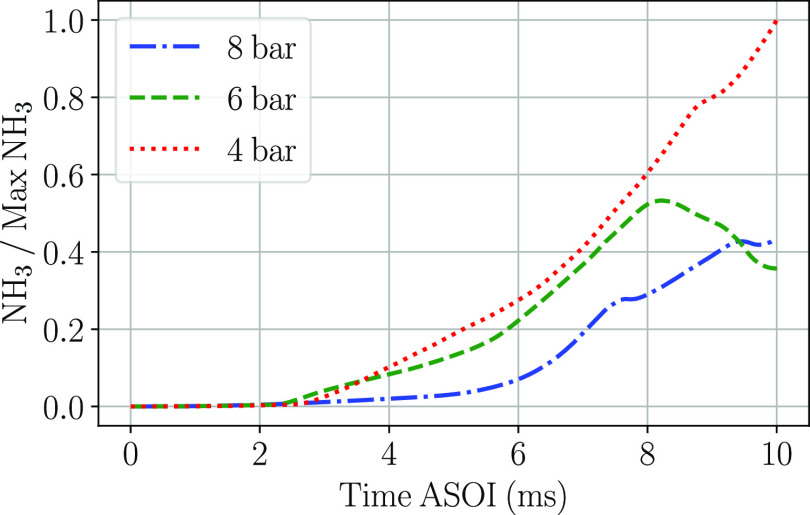
Evolution of the ratio of NH_3_ to the maximum NH_3_ detected at the three injection pressures simulated.

If the amount of ammonia generated at the three injection pressures for the time simulated is analyzed, the curves from Figure 21 are obtained. The amount of ammonia has been normalized with respect to the maximum ammonia amount found within the three mentioned injection pressures. The minimum injection pressure shows the greatest amount of NH_3_ found, while for 6 and 8 bar simulations, the maximum ratio is found at 8 and 10 ms, respectively. The lower injection velocities associated with the lowest injection pressure increases the droplet residence time within the domain to undergo the thermolysis process. The higher injection pressures on the other hand show a large amount of droplets vanishing from the domain through the lower walls; therefore, from a certain instant, the amount of NH_3_ starts to decrease. This is the reason why almost no differences were found at the droplet diameter PDF curves for 6 and 8 bar of injection pressure, while for 4 bar of injection pressure, slightly greater differences could be observed.

## Conclusions

The present work developed a chemical model capable of predicting urea degradation into ammonia. This would allow a better understanding of the processes that undergo the UWS spray from its injection until the droplets evaporate and transform into ammonia and its byproducts. The chemical model has been validated against existing experimental data and has been applied to recreate the results of an in-house experimental facility. From this study, the following conclusions could be extracted:

- The proposed chemical model accurately predicts the (NH_2_)_2_CO to NH_3_ conversion efficiency through the thermolysis (eq 1) and hydrolysis (eq 2) mechanisms at temperatures below 623 K for the different gas velocities. With a gas temperature of 673 K, the three velocities show greater amounts of ammonia than expected.
- The main conversion driver being the gas temperature, at low cross-flow gas temperatures (453 K), the effect of implementing a urea degradation model is negligible when it comes to analyzing the droplet size and velocity distributions. This could lead to low deNO_*x*_ efficiency during engine conditions at low exhaust temperatures.
- Two distinct droplet size reduction processes can be distinguished from the mean droplet size evolution curves. These correspond to the water evaporation content of the UWS spray, and later the urea conversion to its products, which happens at a considerably lower rate.
- High injection pressure conditions seem to enhance the droplet breakup, which leads to a faster water evaporation and a higher urea to NH_3_ conversion as seen in the droplet size time evolution and the droplet size gradients. At higher cross-flow gas temperatures, the water evaporation gradient is increased for the three injection velocities, while the evaporation rate during the thermolysis process is quite similar between the two cross-flow temperatures, being slightly higher for the 623 K case.
- Only thermolysis process is detected by means of observing the HNCO and NH_3_ contours as the location spots of HNCO match the location of the spots where NH_3_ is being produced, which indicates that eq 1 is taking place. Lower injection pressures help the thermolysis process to occur as the droplets endure a higher residence time within the computational domain.

The created model stands as a good methodology for predicting the UWS spray and the chemical processes associated with its injection in engine exhaust conditions. Nonetheless, other configurations of exhaust gas velocities and injector orientation should be tested in order find the optimal conditions to maximize the NH_3_ generation after the injection. In addition, introducing realistic exhaust geometries instead of a simplification might activate local turbulence that would enhance the urea degradation process.
